# Imaging the delayed complications of childhood Kawasaki disease

**DOI:** 10.12688/f1000research.73097.1

**Published:** 2022-02-04

**Authors:** Andrew Crean, Lee Benson, Ashish Shah, Kelly Han, John Lesser, Brian W. McCrindle

**Affiliations:** 1 Cardiology, University of Ottawa Heart Institute, Ottawa, ON, K1Y 4W7, Canada; 2 Cardiology, Hospital for Sick Children, Toronto, ON, M5G 1X8, Canada; 3 Cardiology, St Boniface Hospital, Winnipeg, Manitoba, R2H 2A6, Canada; 4 Cardiology, Children's Minnesota Hospital, Minneapolis, MN, 55404, USA; 5 Cardiology, Minneapolis Heart Institute, Minneapolis, MN, 55407, USA

**Keywords:** Kawasaki disease, cardiovascular magnetic resonance, CMR, cardiac CT, positron emission tomography, PET, intravascular ultrasound, IVUS, optical coherence tomography, OCT

## Abstract

This review will discuss the long-term complications of Kawasaki disease with a particular focus on imaging surveillance of the coronary arteries in adolescence and adult life. The relative advantages and disadvantages of each modality will be illustrated with practical examples, demonstrating that, in many cases, a multimodality imaging strategy may be required.

## Introduction

Kawasaki disease (KD) is a systemic vasculitis of unknown etiology. Coronary arterial complications, both in acute or chronic phase of the illness, result in high morbidity and mortality.

Vascular remodeling characterized by fibroblastic proliferation and matrix metalloproteinase deposition results in progressive fibrosis and, eventually, stenosis. ^1^ The flow pattern in an aneurysmal segment is characterized by recirculation, reduced wall shear stress and stasis, whereas there is flow acceleration through stenotic lesions. ^2^ Such flow alterations result in platelet activation, which, in the presence of endothelial dysfunction, creates a highly thrombogenic milieu, and may result in thrombotic occlusion of the coronary arteries. ^3^

Although aneurysmal coronary artery dilatation is seen initially in pediatric acute KD, the subsequent complications of aneurysm thrombosis, distal embolism and infarction, or inter-aneurysm coronary artery stenosis, are more often seen much later in life. As a result, the sufferer of childhood KD will require life-long surveillance throughout adulthood in those cases where coronary artery dilatation fails to regress in the convalescent period. This review will describe these complications and offer suggestions to guide the selection of imaging modality ( Table 1), through discussion of each in turn.

**Table 1.** Relative strengths and weaknesses of commonly-used imaging modalities for Kawasaki disease.

	Echo	SPECT	PET	CT	CMR	Angiography	IVUS/OCT
Coronary artery calcification or plaque	-/+	-	+	+++	+	++	++++
Coronary artery aneurysms	+	-	-/+	+++	++	+++	++
Coronary artery stenoses	-/+	-	-	+++	++	++++	++
Thrombus within aneurysms	+	-	-	++++	+++	++	++
Myocardial infarction	+	++	+++	++	++++	+	-
Myocardial perfusion	+/++	++	++++	+/++	+++	-	-

SPECT, single photon emission computed tomography; CT, computed tomography; PET, positron emission tomography; CMR, cardiovascular magnetic resonance; IVUS, intravascular ultrasound; OCT, optical coherence tomography.

### Echocardiography

**Figure 1.**
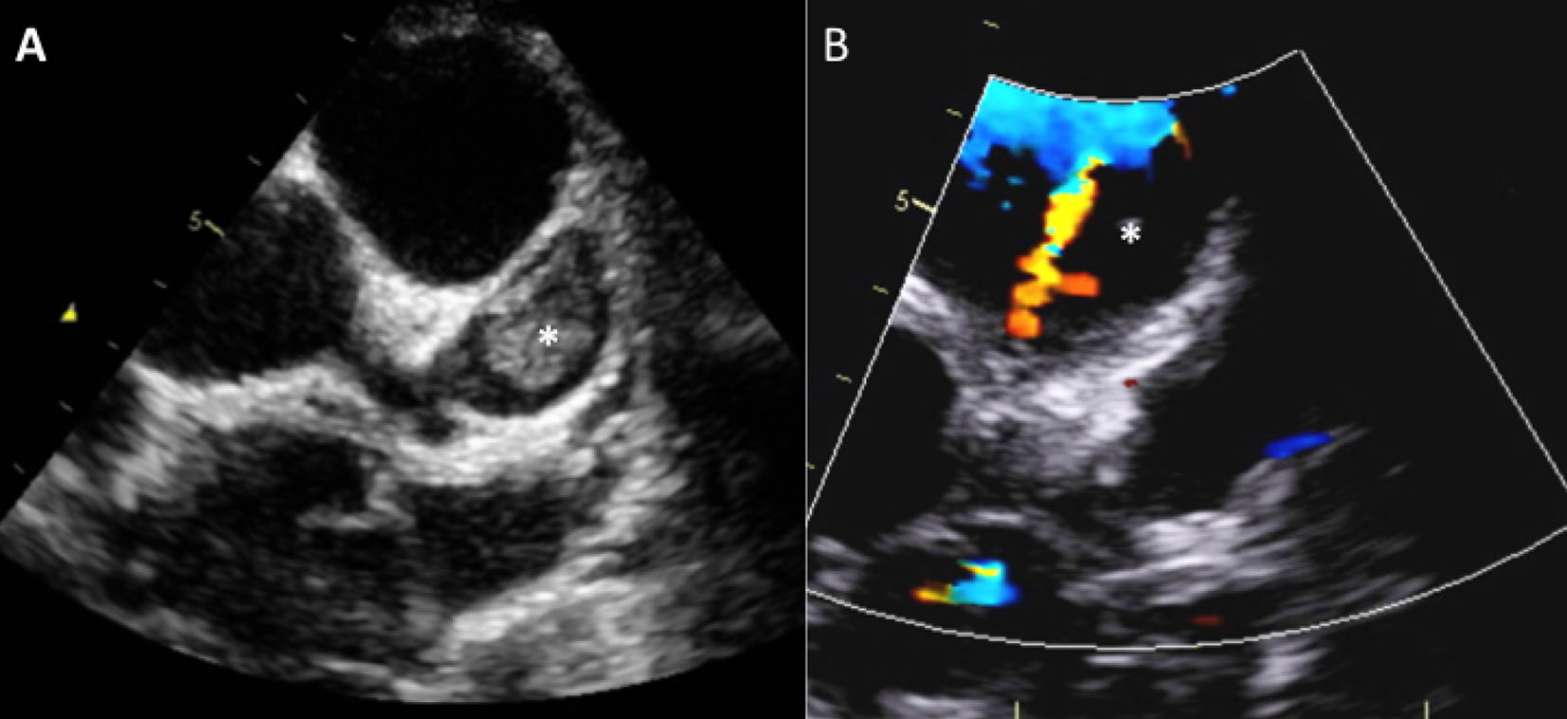
Evidence of acute thrombus in LAD seen on echocardiography. A) Thrombus (asterisk) within the proximal portion of the large LAD aneurysm. B) Accelerating narrowed diastolic flow jet around the peripheral rim of the thrombus. LAD, left anterior descending.

Echocardiography is always the initial tool in the assessment of the patient with Kawasaki disease. Young children generally have excellent echocardiographic windows with the proximal portions of the coronary arteries being fully visualized. Imaging features to note include coronary artery dilatation (which on occasion may be subtle); ectasia and lack of distal tapering; frank aneurysmal formation; and perivascular echogenicity or “brightness” due to inflammation of the arterial wall (in acute presentations). Aneurysms may be isolated or segmental with intervening ‘skip areas’ of normal vessel and they may be either saccular or fusiform in shape. Proximal aneurysms can be accurately measured and evaluated for internal thrombosis or occlusion ( Figure 1).

The examination should also include a formal evaluation of cardiovascular function. Acutely unwell patients frequently have an associated myocarditis and may have wall motion abnormalities on 2D or 3D imaging. Associated pericarditis may present with pericardial effusion. Myocardial deformation tracking using speckle tracking may demonstrate improvement early in the recovery period, ahead of global measures such as ejection fraction, ^4^ but may also remain abnormal in the long term, especially in more severely affected patients. ^5^

Acute coronary artery thrombosis, usually the result of *in situ* thrombosis within an aneurysm, may result in myocardial infarction or stunning and profound regional wall motion abnormality. Similarly, distal embolization of thrombus within an aneurysm may lead to myocardial infarction, wall motion abnormality and eventually regional thinning.

Valvular involvement in Kawasaki disease is reported but is rarely a prominent feature. Nonetheless the presence of severe mitral regurgitation should raise the question of underlying myocardial ischemia and secondary papillary muscle dysfunction.

## Nuclear perfusion imaging

**Figure 2.**
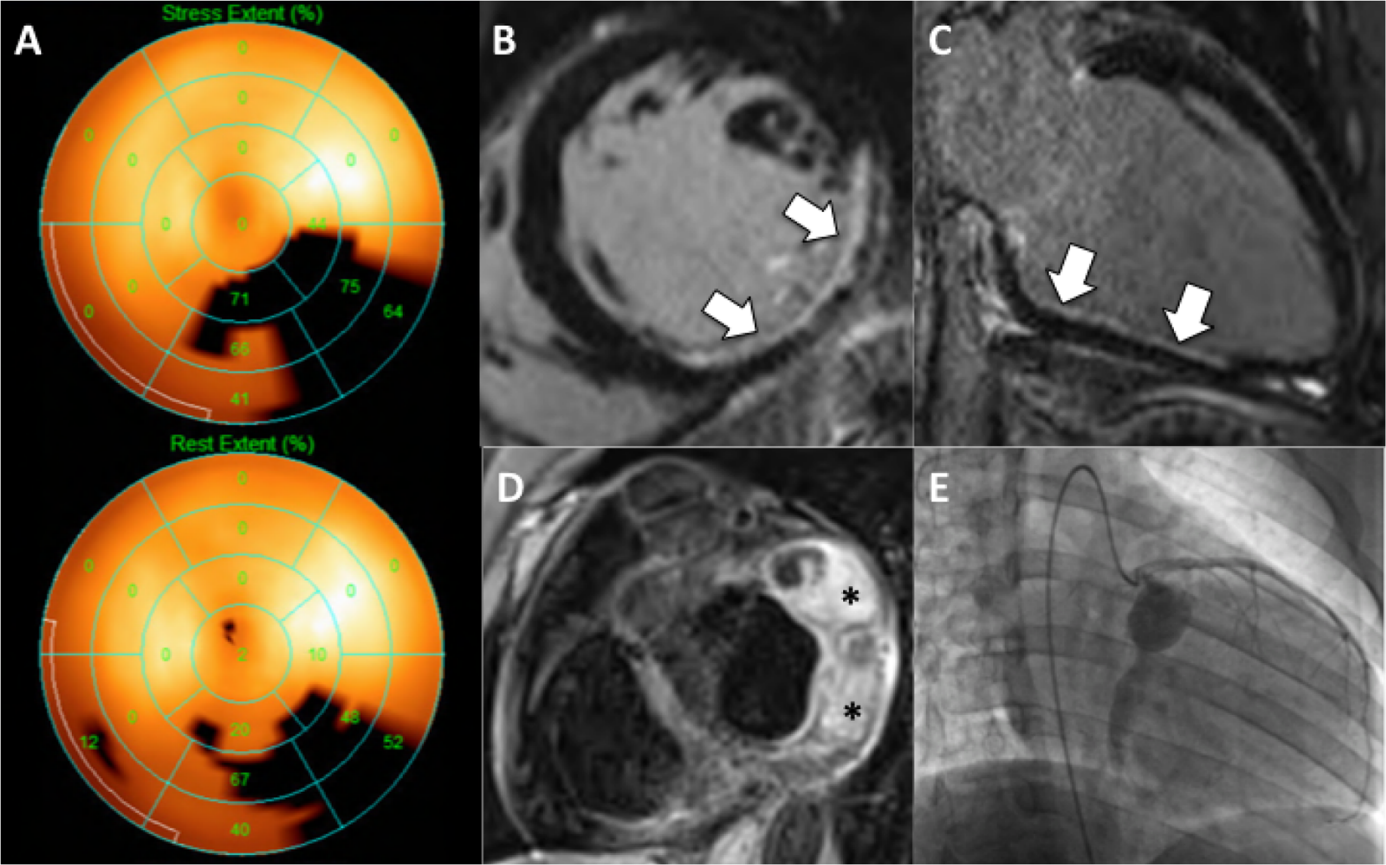
Circumflex territory infarct. A) Nuclear SPECT perfusion study demonstrating a largely fixed infero-lateral defect on polar map. B,C) Late gadolinium enhancement reveals a non-transmural scar (white arrows) in the same area. D) Black-blood CMR shows circumflex aneurysms with thrombus (asterisks). E) Angiogram confirms Cx aneurysms with poor distal flow.

Nuclear medicine techniques were, for a long time, the mainstay of non-invasive imaging in Kawasaki disease for the detection of flow-limiting lesions and infarction ( Figure 2). The ready availability of SPECT equipment together with the ability to combine perfusion and treadmill imaging has made this, historically, the most widely used approach to following patients in the chronic phase, both before and after coronary artery bypass surgery. ^6^ There are no data in a sufficiently large sample from which to derive diagnostic accuracy estimates of stress SPECT; however, the ability of young people to exercise to high levels together with (generally) lower body mass index, and hence less photon attenuation, compared with the elderly atherosclerotic population, may lead to fewer non-diagnostic or false positive studies. Nonetheless, there are strong arguments for following younger patients by alternative means that do not expose them recurrently to ionizing radiation, such as stress echo and stress CMR.

Nuclear cardiology has, however, provided insights into Kawasaki disease, particularly from positron emission tomographic (PET) imaging - which can provide higher accuracy for disease detection than SPECT perfusion imaging, and with lower radiation exposure. ^7, 8^ It has been known for a number of years that coronary endothelial function is abnormal following Kawasaki disease. One early paper examined coronary vasodilatory function in patients who had had Kawasaki disease but without any overt coronary involvement, and compared resting and stress flow to that in normal age-matched controls. Resting flow, measured using ^13^N-ammonia, was comparable in both groups, but interestingly the Kawasaki group had lower levels of hyperemic stress flow (and hence reduced flow reserve) despite ostensibly normal coronary arteries, possibly due to increased microvascular dysfunction. ^9^ Hauser *et al.* demonstrated similar findings in children with normal epicardial arteries after the onset of Kawasaki disease. ^10^ These observations were extended by Furuyama *et al.* who demonstrated similar findings, using ^15^O PET, in Kawasaki patients with regressed aneurysmal dilatation; but, using the cold pressor test to generate hyperemia, additionally showed impairment of the endothelial-dependent pathway. ^11^ A more recent small study demonstrated even greater impairment of endothelial-dependent vasodilatation (as assessed by cold pressor test) in patients with frank aneurysmal dilation compared to those with regressed aneurysms, arguing for a gradation of endothelial damage across the spectrum of disease. ^12^

Rubidium-82 generators have now been available for several years and this tracer might be an additional clinical tool to assess further the myocardial blood flow metrics on dynamic PET. There are, as yet, no reports of myocardial blood flow assessment using ^82^Rb in Kawasaki disease.

## Cardiovascular magnetic resonance (CMR)

CMR is arguably the single most useful non-invasive modality for following patients from the age of 8 years and upwards. The strength of CMR lies in the multiplicity of techniques available for assessing structure, function, and tissue characterization.

**Figure 3.**
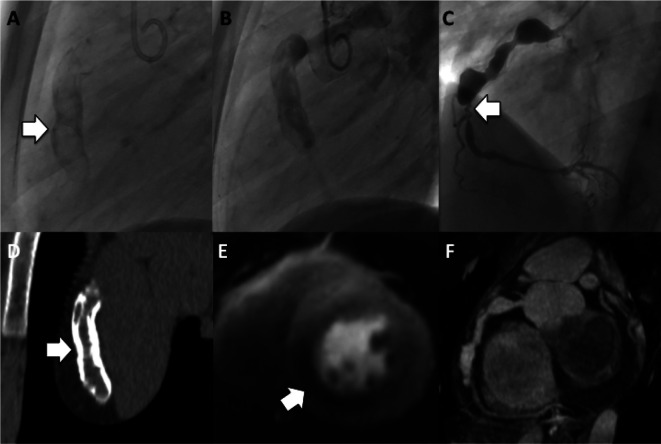
Stress perfusion CMR in right coronary artery stenosis. A,D) Calcific cast of RCA (arrow) is noted prior to contrast injection. B,C) Note that the contrast column does not touch the vessel edge outlined by the calcium, consistent with the presence of laminar thrombus. A focal tight mid RCA stenosis is present (arrow). E) Area of hypoperfusion (arrow) in the basal infero-septum on dipyridamole stress CMR. F) Whole heart coronary MRA of RCA corresponding to image in C – there is good correlation for aneurysm morphology, but note that the RCA distal stenosis is less well visualized due to inferior spatial resolution of CMR compared to conventional angiography.

**Figure 4.**
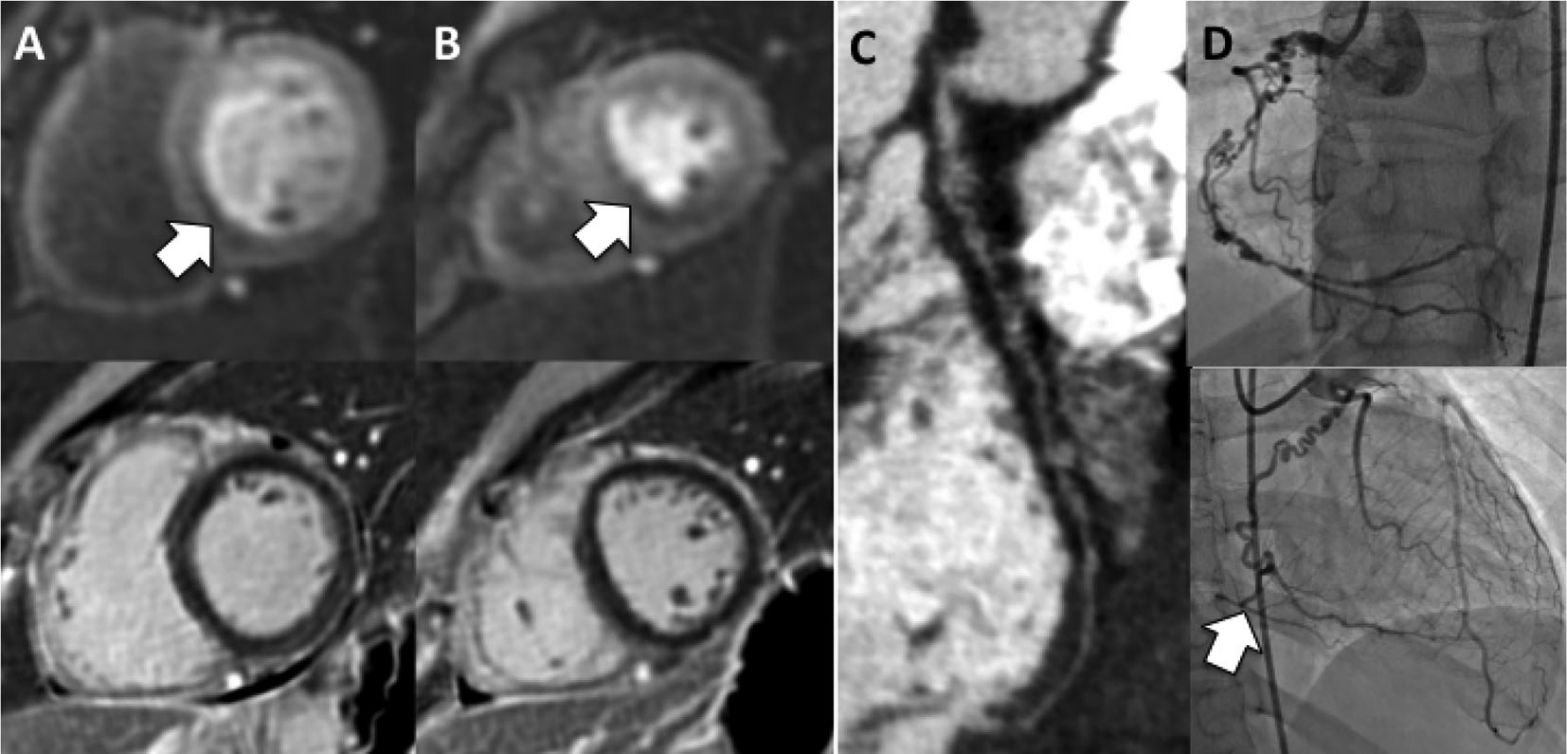
Stress perfusion CMR in the context of collateralization. A,B) A zone of subendocardial hypoperfusion is evident in the RCA territory (arrows, top panels). Note the lack of underlying scar on the corresponding LGE images (bottom panels). C) Cardiac CTA shows a long segment of irregular and hypo-enhanced RCA suspicious for diffuse disease with high-grade stenoses. D) Coronary angiography in fact shows RCA occlusion with extensive bridging collaterals from proximal to distal RCA forming a ‘woven’ vessel. Further collateralization is seen from the Cx to the distal RCA (arrow, bottom panel). Extensive collateralization like this may reduce the size of the perfusion defect (as here) and the true ischemic burden can be difficult to judge. This young woman wished to get pregnant and went ahead without revascularization after Bruce protocol treadmill stress echo where she managed 13 METS of activity without symptoms, ECG changes or wall motion abnormality.

Stress perfusion MRI is increasingly being performed in congenital and inflammatory coronary conditions. ^13– 15^ The overall diagnostic accuracy has not been adequately established for non-atherosclerotic coronary conditions, but based on data from the CEMARC study is likely to be higher than for SPECT. ^16^ Most centers currently interpret stress CMR images qualitatively ( Figure 3). Interestingly, quantitative perfusion CMR has demonstrated abnormalities of microvascular function, which is entirely concordant with the available PET literature. ^16^ Nonetheless, qualitative perfusion imaging in Kawasaki Disease can be problematic, as extensive collateral networks may form and, even in the setting of total occlusion, perfusion can appear qualitatively normal. It is for this reason that we usually combine cardiac CT and CMR in our practice ( Figure 4).

It should be recognized that inducible perfusion defects may be evident even after coronary artery bypass surgery, as incomplete revascularization is often inevitable.

**Figure 5.**
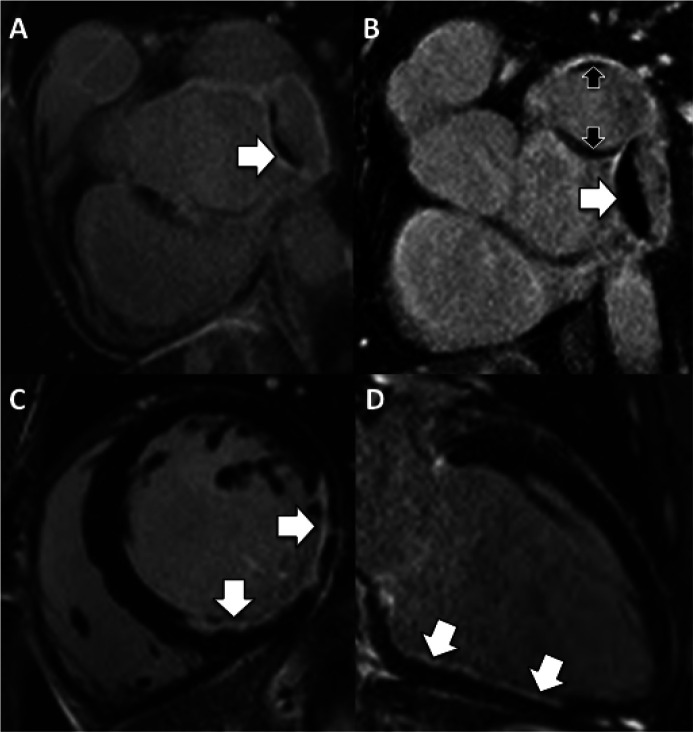
Progressive thrombus deposition in Kawasaki aneurysms. A) Late gadolinium enhancement (LGE) acquired in 2010 demonstrates a small rim of thrombus (arrow) in the more distal of 2 sequential Cx aneurysms. B) Repeat in LGE in 2012 reveals increased thrombus burden in this aneurysm (white arrow) and new layers of thrombus in the more proximal aneurysm (black arrows). The patient had not been compliant with anticoagulation during this period. C & D) Mid ventricular short-axis and 2-chamber LGE sequences demonstrate partial thickness infarction (arrows) in the basal to mid inferior and inferolateral walls extending into the inferoseptal wall related to embolism into this dominant circumflex coronary artery.

Late gadolinium enhancement (LGE) imaging provides clear images of scarred and infarcted segments due to prior coronary occlusion and/or embolism ( Figure 5). The technique is recognized as being highly sensitive for even very small volumes of scar.

Less well-appreciated is the value of LGE imaging for detecting intra-coronary thrombus. Patients with giant aneurysms have altered rheology and altered wall characteristics; furthermore, abnormal shear forces may adversely affect platelets. Therefore all three components of Virchow’s triad are often present. Large occlusive thrombus is rarely a difficult diagnosis since the presentation is usually one of acute myocardial infarction. Smaller amounts of thrombus can be quite challenging to visualize and are often missed by echo, as they usually line the walls of an aneurysm without causing much reduction in internal diameter or disrupting flow. The LGE technique provides excellent visualization of ventricular thrombi due to the low signal returned from thrombus contrasting well with the grey/white signal from the coronary blood pool ( Figure 5).

## Cardiac CT

Cardiac CT is a useful and reasonable method for ‘staging’ new referrals to the adult Kawasaki clinic. In our clinic it is a baseline test to establish the presence and extent of aneurysmal disease and stenosis and may act as a gatekeeper to subsequent coronary angiography.

**Figure 6.**
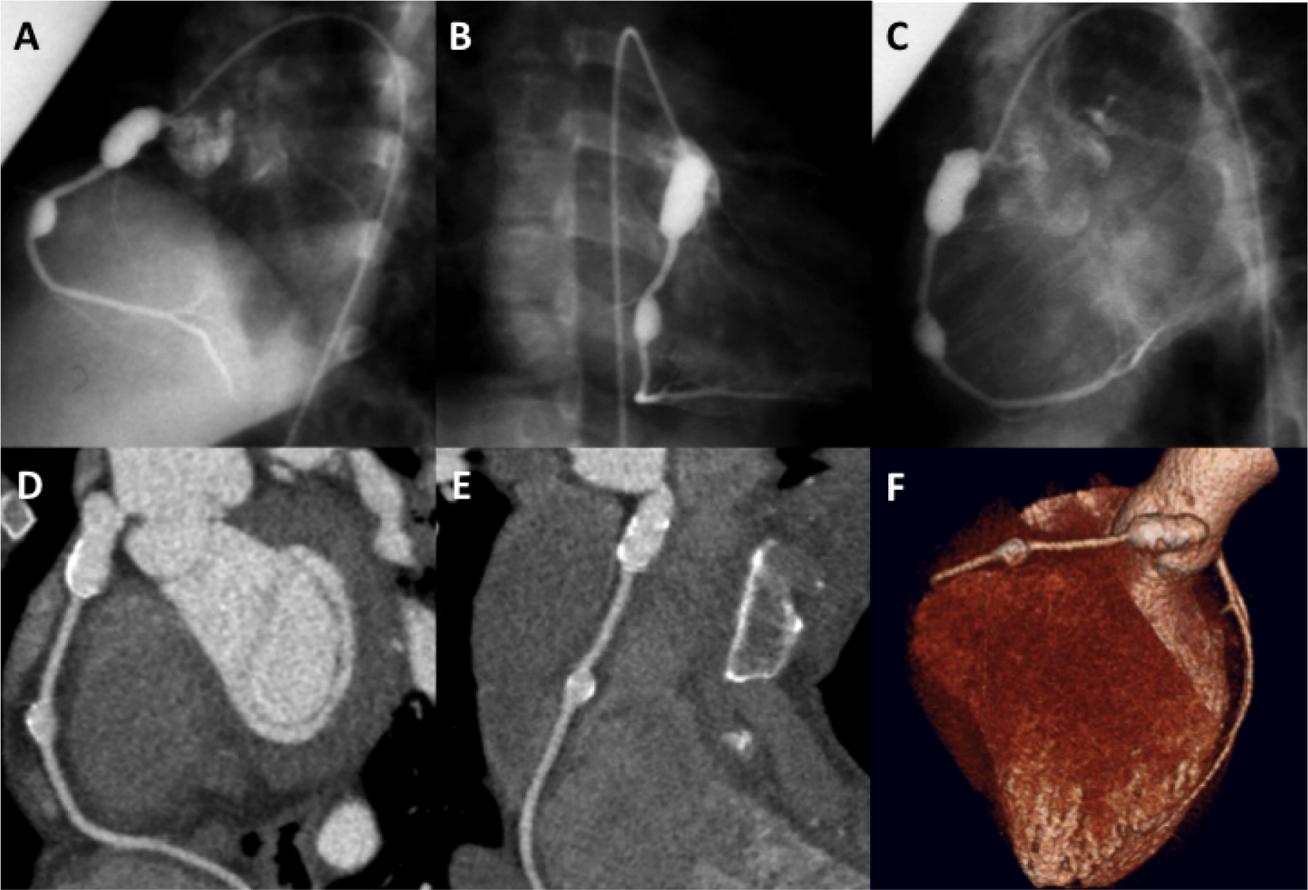
Cardiac CT vs catheter correlation. A-C) Conventional angiographic views display 2 giant aneurysms in the RCA. D,E) Curved multiplanar reformats from cardiac CT data set demonstrate good correlation and more clearly depict wall calcification and lack of mural thrombus. F) Volume-rendered image from a cardiac CT data set represents an alternative visual method for displaying CT findings.

The major advantages of cardiac CT are its rapidity, spatial resolution, and sensitivity for calcium. Modern scanners can complete acquisition of the target anatomy in a few seconds (or less) making CT very suitable for the restless or claustrophobic. Spatial resolution is in the region of 0.5 mm for most scanners which is adequate for the depiction of aneurysms, thrombus and normal or ectatic coronary segments. Clinically useful coronary coverage is almost invariably greater by CT than it is by echo ^17^ and good correlation with conventional coronary angiography has been demonstrated ^18^ ( Figure 6).

**Figure 7.**
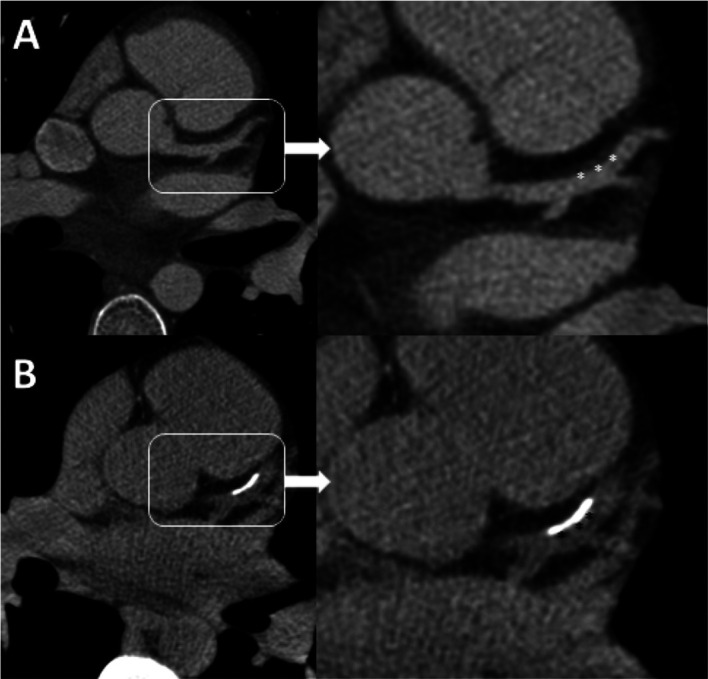
Progression of plaque. A) Initial coronary CT demonstrates subtle soft plaque/vessel wall thickening (white asterisks) in the proximal LAD. B) Calcium score, 3 years later, now shows new overlying calcification (black asterisks) implying mineralization secondary to chronic low-grade inflammation. Statin therapy should be considered for all KD patients although the level of evidence for treatment is weak.

Unlike CMR, cardiac CT is exquisitely sensitive for even small amounts of calcium. Calcium seems to be mainly associated with aneurysms and in patients above the age of ten. ^19^ It is uncertain whether this matters, as it remains unclear whether Kawasaki patients have increased propensity to develop accelerated atherosclerotic lesions as they get older. Occasionally, however, calcification may occur at the site of minimal coronary dilatation, presumably secondary to healing following immune-mediated vasculitis ( Figure 7).

Cardiac CT may also be used to identify intra-aneurysmal thrombus. ^20^ A delayed phase data set acquired roughly 60 seconds after injection is required in addition to the arterial phase coronary acquisition, to permit full mixing of iodine with blood, which may be delayed in larger aneurysms, leading to filling defects and pseudo-thrombus ( Figure 10).

Myocardial scar, due to infarction or embolism, may also be recognized when late iodine enhancement imaging is performed with care. This is, however, an area where CMR retains superiority.

**Figure 8.**
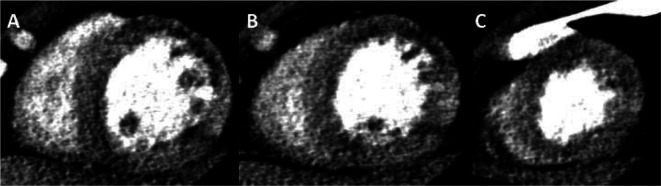
Dipyridamole stress CTA. A-C) Basal, mid and apical short axis reconstructions of a stress CT data set demonstrating an LAD territory perfusion defect in a Kawasaki patient with LAD aneurysm and prior occlusion.

Finally vasodilator stress CT has demonstrated incremental benefit in identification of flow-limiting disease in adult atherosclerotic disease. ^21^ There are no published data on this technique in Kawasaki disease, though it could be attractive in theory due to benefits derived from perfectly co-registered anatomy and perfusion data sets ( Figure 8).

## Cardiac catheterization

### Coronary angiography

**Figure 9.**
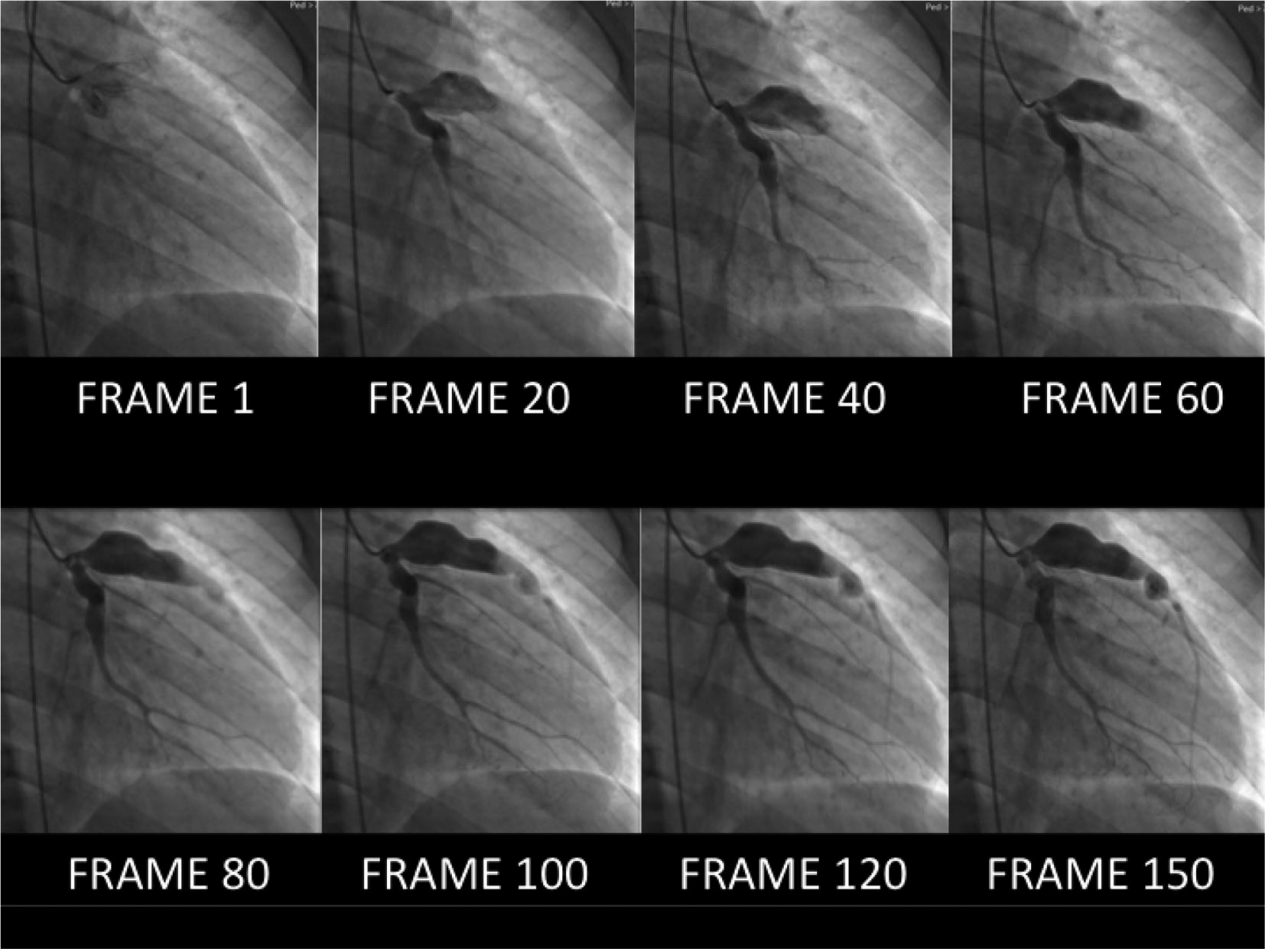
Coronary stasis. Multiple frames are shown from the left coronary injection of a conventional angiogram. A large saccular proximal LAD aneurysm is outlined. Note the extreme delay before contrast fully opacifies the distal LAD at frame 150 from the start of injection. It is this intra-coronary stasis that accounts for the high thrombotic risk in this patient population, even on anticoagulants.

**Figure 10.**
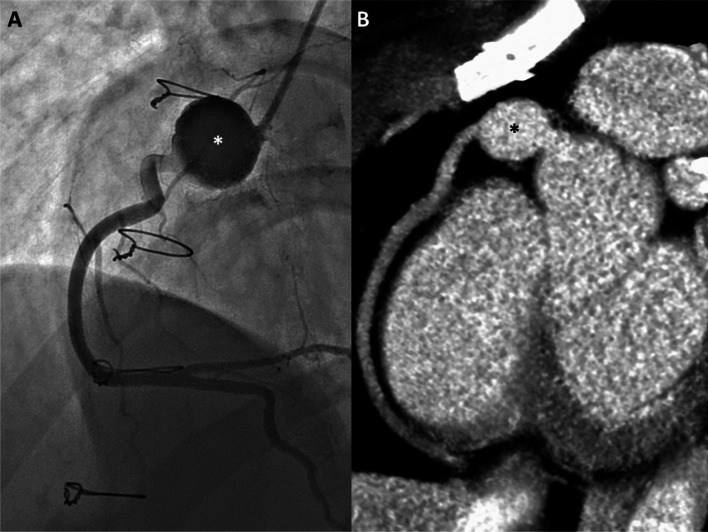
Pseudo-thrombus at catheterization. A) Right coronary injection demonstrates a large proximal aneurysm with an apparent central oval lucency (white asterisk). This was interpreted as a ball-valve thrombus in this young man with chest pain symptoms. B) Same-day coronary CT acquired 1 minute after injection shows completely uniform iodine intensity within the aneurysm (black asterisk), confirming the absence of any real thrombus – indicating that the catheter finding was the result of swirling flow and incomplete mixing of blood and iodine immediately after injection.

Angiography remains the tool of choice in symptomatic patients or those with equivocal non-invasive imaging. As a ‘real-time’ injection of contrast is made, the degree of sluggish or swirling flow is much more obvious than with cross-sectional techniques ( Figure 9). Nonetheless, we have occasionally seen cases where swirling flow was misinterpreted as ball valve thrombus in a proximal giant aneurysm ( Figure 10).

### Coronary intervention

Thrombotic occlusion of the coronary artery is a feared complication in patients with KD, not only in the acute phase of illness, but also many years later. In a case series of 50 adult patients, thrombotic occlusion of the aneurysmal segment was the most common complication. The majority of the patients were treated with balloon angioplasty and/or thrombolytic use; rotational atherectomy and stenting were used in only one patient each. IVUS examination in some of these patients demonstrated thickened intima, as expected, but no atherosclerotic changes were noted. ^22^

Balloon angioplasty, even with high-pressure balloons, is unlikely to be successful in adult patients, due to presence of dense fibrosis. ^23^ Such an approach can result in neo-aneurysm formation that has the potential to result in stent malapposition. Similarly, because of variation in vessel caliber, due to interspersed aneurysm and stenotic lesions, achieving satisfactory stent apposition can be challenging, ^24^ and malapposed stent struts are an independent risk factor for stent thrombosis.

Percutaneous coronary intervention in patients with KD should be guided by intra-coronary imaging that can help differentiate between densely calcified lesions versus fibrotic ones, as rotational atherectomy can be successfully used to treat the former, whereas use of a cutting balloon would be preferred in the latter. ^25^

### Fractional flow reserve (FFR)

Visual assessment of coronary angiography has inherent limitations. FFR measurement has been validated and extensively used to assess physiologic significance of obstructive coronary artery disease. The presence of sequential narrowings in the same artery results in a cumulative impact on myocardial perfusion. Interestingly, not only coronary stenosis, but also aneurysmal dilatation results in ‘pressure loss’ due to turbulence, which may contribute towards worsening of coronary perfusion. ^26, 27^ In patients with KD, whose coronary arteries demonstrate diffuse stenosis and aneurysmal dilatation, visual assessment by angiography alone is inadequate, and FFR should be considered. One caveat though is that FFR assessment relies on vascular endothelial response to variety of vasoactive substances. Patients with KD are known to have endothelial dysfunction that can affect peak hyperemia, although the impact of such dysfunction on FFR measurement is not known.

### IVUS, OCT

Intravascular ultrasound (IVUS) uses piezo-electric transducer or electronic phased array to sends out sound signals that are reflected by the tissue it passes through, according to their acoustic properties. IVUS examination of the coronary arteries, many years after an acute episode of KD often demonstrates thickened intima-media complex, and diffuse calcification at the sites of persisting or regressed aneurysms, but atherosclerotic changes are not usually seen. ^28^

**Figure 11.**
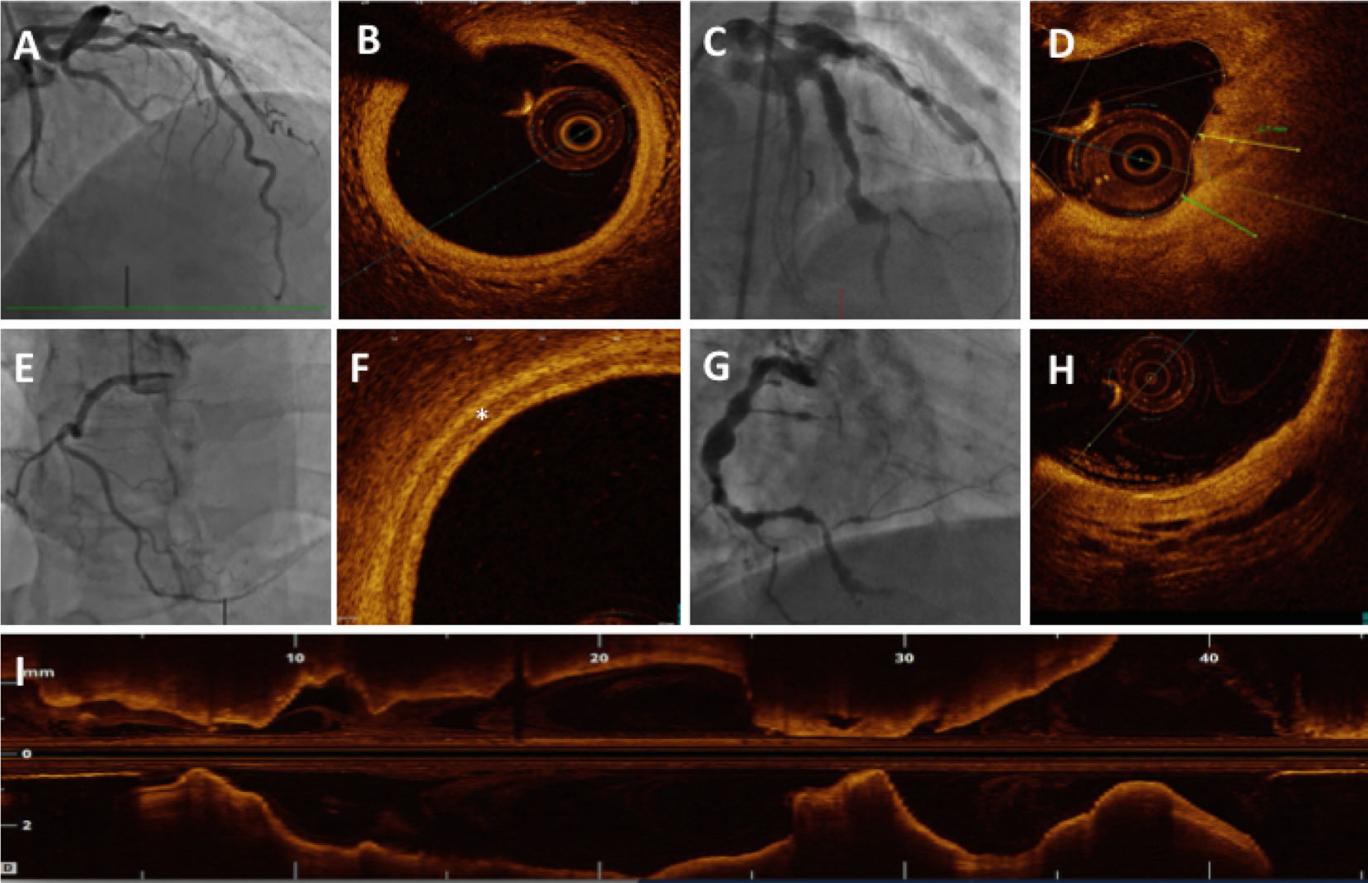
Optical coherence tomography. (A & E) Coronary angiogram demonstrating mild atherosclerotic disease. (B & F) OCT examination of these coronary arteries demonstrating normal intima-media where * demonstrates muscular media. (C & G) Angiography demonstrating coronary arteries many years after Kawasaki disease. OCT examination of these arteries demonstrates destroyed intima-media structure that is replaced by thick fibrosis (D) and disintegrated muscular layer (H). (I) Longitudinal reformatted OCT image of the same coronary artery demonstrated aneurysmal and stenotic segments along its length.

Optical coherence tomography (OCT) measures backscatter of the light (optical echos), and offers the highest possible resolution images below 10 μm ( Figure 11). OCT examination allows higher definition image acquisition of a fibrous cap (thickness, cellular infiltration and erosion), necrotic core, calcified lesions, plaque rupture, dissection plane, and stent apposition. ^29, 30^ These images are much clearer than the ones acquired with IVUS. OCT examination provides detailed tissue information, comparable with the histopathological examination of the coronary arteries. ^31^ However, higher resolution comes at a cost of limited penetration; OCT may not be an imaging modality of choice in large or giant aneurysms.

**Figure 12.**
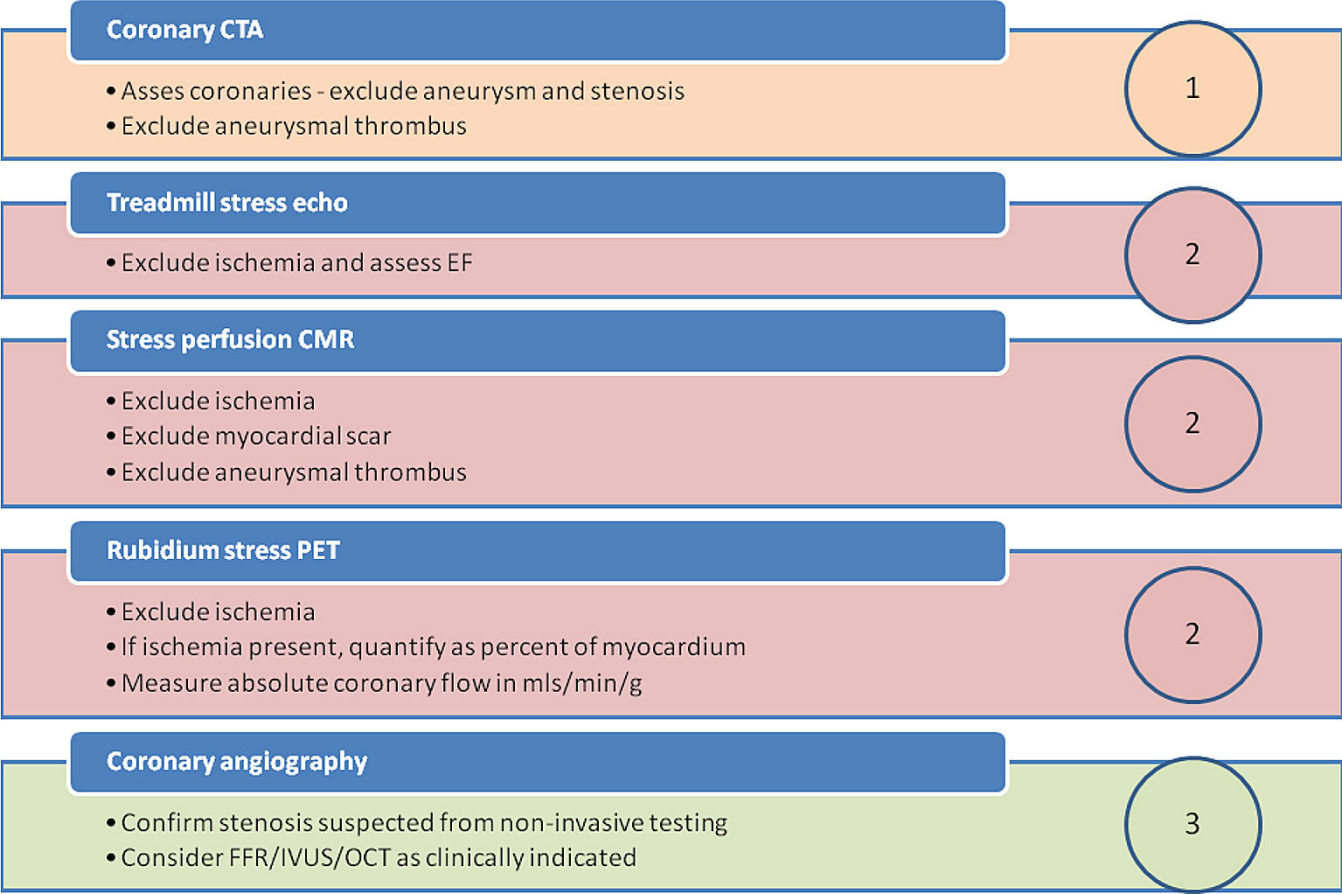
A hierarchical approach to testing in complicated adult Kawasaki disease. Newly referred adults - with a history of Kawasaki disease in childhood - are staged with up to 3 levels of investigation. **Level 1:** Coronary CTA is performed first. If there is no evidence of coronary involvement (or no worse than only mild dilatation), no further investigation is required. If there is evidence of aneurysm or stenosis further testing is required. **Level 2:** The precise test chosen should depend upon availability and local expertise. If embolic infarction from aneurysmal thrombus is suspected, then CMR is often the most revealing test. Where the selected level 2 test is equivocal, we often proceed to an alternative level 2 test for confirmation. When there remains residual doubt, or if a level 2 test is positive, we proceed to the next step. **Level 3:** Invasive coronary angiography with or without hemodynamic assessment of severity of stenosis by FFR. While OCT and IVUS may be useful to assess stent deployment, percutaneous coronary intervention is often a poorer choice than bypass surgery for the majority of Kawasaki patients, given the variability in cross-sectional diameter of involved segments.

## Conclusions

Kawasaki disease patients with known coronary involvement should be followed carefully throughout adult life. A single approach is unlikely to be able to reliably demonstrate all of the possible coronary and cardiac complications that can result from this disease. Appropriate use of imaging allows for early detection of complications with subsequent surveillance and may reduce subsequent morbidity and mortality.
