# Supplementary material for: Imaging the delayed complications of childhood Kawasaki disease
Source: F1000Res. 2022 Feb 4;11:147. [Version 1] doi: 10.12688/f1000research.73097.1 (PMC10036956; doi:10.12688/f1000research.73097.1)
Supplement: Peer review history

# Peer review: Reviewer response for version 1

**DOI:** 10.5256/f1000research.76721.r163155

**Published:** 2023-03-23

**Authors:** Rakesh Kumar Pilania

**Affiliations:** 1 Department of Pediatrics, Institute of Medical Education and Research, Chandigarh, 160012, India

Crean et al. have submitted their review of imaging the delayed complications of childhood KD. Authors have provided comprehensive review on the subject. However, I have few comments.  

  

1. Echo:
  1. Author should define difference between dilatation, ectasia, and aneurysms clearly for benefit of readers. Authors can add images of echocardiography differentiating among these terminologies.
  2. Though ECHO may be an initial modality of imaging at presentation, however as the child grows ECHO may be challenging due to smaller acoustic window, inability to demonstrate the CAAs in mid and distal segments and LCx. complications like thrombus. stenosis or mural calcifications.
  3. Ectasia is confusing terminology and nowadays not very common in use.
  4. Limitations of Echo need to be talked upon, especially identification of distal aneurysms, thrombosis, stenosis, calcifications.
2. Nuclear scan: Role of nuclear imaging is need to be balanced in current era where CTCA, ECHO and CMR would be more important imaging.
3. CMR: CMR is most useful imaging modality in the late phase of disease. Authors have detailed almost very aspect of that. However, Article is silent on MRCA. Role of MRCA needs more literature search and its role and comparison with CTCA.
4. Cardiac CT: 
  1. CTCA is definitely an imaging of choice for evaluation in an adult with history of KD.
  2. It would be important to highlight the strengths of CTCA in comparison to 2D-Echo in identification of distal coronary artery involvement, entire course of coronaries, LCX, complications, stenosis etc.  Singhal M et al. Distal coronary artery abnormalities in Kawasaki disease: experience on CT coronary angiography in 176 children ^1^.
  3. Radiation exposure optimization protocol.
  4. Authors describe CTCA as gate keeper modality: to be elaborated further
  5. Detection of calcification need to be more highlighted. Chakraborty R, et al. Coronary arterial abnormalities detected in children over 10 years following initial Kawasaki disease using cardiac computed tomography ^2^. 
5. Cardiac cath: authors have identified very specific indications for cath angio in a given patient with KD.
6. Authors have provided imaging approach in a complicated patient with KD. This figure is not cited in text. It would be better to expand on that before conclusions section with separate subheading. Authors have given equal weightage to rubidium stress PET to stress perfusion CMR and treadmill stress ECHO. As rubidium stress PET would have associated exposure with radiation exposure. Author can justify their approach.
7. Minor errors: Author can use consistently use abbreviation for Kawasaki disease as KD throughout the manuscript. Please check spacing and abbreviations throughout the manuscript.  

‘

Is the review written in accessible language?

Yes

Are all factual statements correct and adequately supported by citations?

Yes

Are the conclusions drawn appropriate in the context of the current research literature?

Partly

Is the topic of the review discussed comprehensively in the context of the current literature?

Partly

Reviewer Expertise:

Kawasaki disease

I confirm that I have read this submission and believe that I have an appropriate level of expertise to confirm that it is of an acceptable scientific standard, however I have significant reservations, as outlined above.

## References

[1] : Distal coronary artery abnormalities in Kawasaki disease: experience on CT coronary angiography in 176 children. *Rheumatology (Oxford)* .2023;62(2) : 10.1093/rheumatology/keac217 815-823 10.1093/rheumatology/keac217 35394488

[2] : Coronary arterial abnormalities detected in children over 10 years following initial Kawasaki disease using cardiac computed tomography. *Cardiol Young* .2021;31(6) : 10.1017/S1047951121000020 998-1002 10.1017/S1047951121000020 33504398

# Peer review: Reviewer response for version 1

**DOI:** 10.5256/f1000research.76721.r154822

**Published:** 2022-12-23

**Authors:** Kai Sheng Hsieh

**Affiliations:** 1 Children's Hospital and School of Medicine, China Medical University, Taichung, Taiwan

This manuscript discussed the imaging modalities in the late phase of childhood Kawasaki disease. The authors thoroughly reviewed the role of echocardiography, nuclear perfusion imaging, positron emission tomography, computed tomography, cardiovascular magnetic resonance angiography, coronary artery angiography and intervention, coronary fractional flow low reserve, intravascular ultrasound, and optical coherence tomography (SPECT/PET/CT/CMRAngiography/CAG/CFFR/IVUS/OCT). The review is comprehensive and informative. The quality of various figures also are good. The authors also list a table to display the relative strengths and weaknesses of commonly used imaging modalities for Kawasaki disease. The content of this manuscript is thus of sufficient quality and I will suggest to index this manuscript as its current form and no revision is necessary.

Is the review written in accessible language?

Yes

Are all factual statements correct and adequately supported by citations?

Yes

Are the conclusions drawn appropriate in the context of the current research literature?

Yes

Is the topic of the review discussed comprehensively in the context of the current literature?

Yes

Reviewer Expertise:

Pediatric Cardiology, echocardiography, Pediatric cardiac imaging

I confirm that I have read this submission and believe that I have an appropriate level of expertise to confirm that it is of an acceptable scientific standard.

# Peer review: Reviewer response for version 1

**DOI:** 10.5256/f1000research.76721.r136684

**Published:** 2022-06-10

**Authors:** Diana van Stijn

**Affiliations:** 1 Department of Pediatric Immunology, Rheumatology, and Infectious Diseases, Emma Children's Hospital, Amsterdam UMC, University of Amsterdam, Amsterdam, The Netherlands

Andrew *et al.* have submitted "Imaging the delayed complications of childhood Kawasaki disease". The aim of this paper is to discuss imaging modalities used in Kawasaki disease patients during adolescence and adult life. First of all we would like to appraise the enormous effort in performing this hands-on review. Knowledge on cardiovascular imaging in adults and young adults with a history of Kawasaki disease is of great importance.

**Minor comments:**

*Overall*

 The authors may consider giving a bit more in-depth background information on the risks that come hand-in-hand with developing CAA after Kawasaki disease (mostly in patients with persistent giant aneurysms). Moreover, it would be nice to add some information on size-dependent long-term risk of having a small, medium and giant CAA. Also, few references are used throughout the article. Although the article discusses very useful expert opinions, it would be nice to add some references with supporting data (from this or other research groups) as well.

*1. Abstract*

 We would strongly recommend using the term "imaging modalities" instead of "imaging surveillance". As imaging surveillance might suggest a hands-on flowchart of the desired imaging over time during follow-up. While the strength of this review lies more in depicting the potential of visualizing pathology with specific imaging modalities.

*2. Introduction*

 “Coronary arterial complications, both in acute or chronic phase of the illness, result in high morbidity and mortality.” This sentence might give the impression that Kawasaki disease has a high morbidity and mortality. Please consider rephrasing this sentence and add absolute numbers and references with the risk of developing persistent CAA and consequent complications (such as thrombosis, myocardial infarction etc.).

*3. Introduction section "echocardiography"*

 The authors mention "perivascular echogenicity or "brightness"" as important imaging features on echocardiography. Despite that early reports considered this as an early sign in KD ^1^, recent papers showed this to be a non-specific finding that can also be found in children without KD ^2^, ^3^. Do the authors consider perivascular brightness an important hallmark in the (young) adult population, or is it as questionable as it is during early childhood?

 “Echocardiography is always the initial tool in the assessment of the patient with Kawasaki disease. Young children generally have excellent echocardiographic windows with the proximal sections of the coronary arteries being fully visualized.” Add references indicating that we don’t miss proximal CAA. The authors might also consider discussing whether (or how often) distal CAA may be missed by echocardiography?

 “Valvular involvement in Kawasaki disease is reported but is rarely a prominent feature. Nonetheless the presence of severe mitral regurgitation should raise the question of underlying myocardial ischemia and secondary papillary muscle dysfunction.” Please add references.

*4. CMR*

 “Nonetheless, qualitative perfusion imaging in Kawasaki Disease can be problematic” A typo: Kawasaki Disease not Kawasaki disease. In the last few paragraphs many statements are made without references, please add references.

*5. Cardiac CT*

 The authors mention something very important for the transition to adult care, namely "staging" new referrals by performing a CT. Would the authors, based on their expert opinion, advise pediatricians/pediatric cardiologists prior to referring a young adult to adult care, to perform a CT?

 “Cardiac CT is a useful and reasonable method for ‘staging’ new referrals to the adult Kawasaki clinic. In our clinic it is a baseline test to establish the presence and extent of aneurysmal disease and stenosis and may act as a gatekeeper to subsequent coronary angiography.” When do authors recommend using cardiac CT in the pediatric population?

 Please consider discussing radiation exposure as a disadvantage of CT as well.

 Several statements are made without references, for example:

 “The major advantages of cardiac CT are its rapidity, spatial resolution, and sensitivity for calcium. Modern scanners can complete acquisition of the target anatomy in a few seconds (or less) making CT very suitable for the restless or claustrophobic. Spatial resolution is in the region of 0.5 mm for most scanners which is adequate for the depiction of aneurysms, thrombus and normal or ecstatic coronary segments.”

*4. Figure 12*

 This figure is of great value, knowing the scope of this article is more towards the value of imaging modalities and not so much surveillance. Could you still elaborate on how often you recommend to perform level 3 imaging? In children we tend to refrain from invasive coronary artery angiography and solely use it when intervention is needed. Therefore we perform follow-up mainly with level 1&2 in KD patients (with coronary artery involvement).

Is the review written in accessible language?

Yes

Are all factual statements correct and adequately supported by citations?

Yes

Are the conclusions drawn appropriate in the context of the current research literature?

Yes

Is the topic of the review discussed comprehensively in the context of the current literature?

Yes

Reviewer Expertise:

Kawasaki disease with focus on cardiovascular pathology and imaging

I confirm that I have read this submission and believe that I have an appropriate level of expertise to confirm that it is of an acceptable scientific standard, however I have significant reservations, as outlined above.

## References

[1] : Is Perivascular Echo Brightness a Reliable Marker of Coronary Arteritis in Acute Kawasaki Syndrome?. *Pediatric Research* .2003;53(1) : 10.1203/00006450-200301000-00139 10.1203/00006450-200301000-00139 12508075

[2] : Examining the Utility of Coronary Artery Lack of Tapering and Perivascular Brightness in Incomplete Kawasaki Disease. *Pediatr Cardiol* .2019;40(1) : 10.1007/s00246-018-1971-z 147-153 10.1007/s00246-018-1971-z 30196380

[3] : Perivascular brightness of coronary arteries in Kawasaki disease. *J Pediatr* .2011;159(3) : 10.1016/j.jpeds.2011.02.029 454-457.e1 10.1016/j.jpeds.2011.02.029 21481416
